# Poisoning of bubble propelled catalytic micromotors: the chemical environment matters

**DOI:** 10.1039/c3nr34213a

**Published:** 2013-03-01

**Authors:** Guanjia Zhao, Samuel Sanchez, Oliver G. Schmidt, Martin Pumera

**Affiliations:** a Division of Chemistry & Biological Chemistry , School of Physical and Mathematical Sciences , Nanyang Technological University , Singapore 637371 , Singapore . Email: pumera@ntu.edu.sg ; Fax: +65 6791-1961; b Institute for Integrative Nanosciences , IFW Dresden , Helmholtzstrasse 20 , D-01069 Dresden , Germany

## Abstract

Self-propelled catalytic microjets have attracted considerable attention in recent years and these devices have exhibited the ability to move in complex media. The mechanism of propulsion is *via* the Pt catalysed decomposition of H_2_O_2_ and it is understood that the Pt surface is highly susceptible to poisoning by sulphur-containing molecules. Here, we show that important extracellular thiols as well as basic organic molecules can significantly hamper the motion of catalytic microjet engines. This is due to two different mechanisms: (i) molecules such as dimethyl sulfoxide can quench the hydroxyl radicals produced at Pt surfaces and reduce the amount of oxygen gas generated and (ii) molecules containing –SH, –SSR, and –SCH_3_ moieties can poison the catalytically active platinum surface, inhibiting the motion of the jet engines. It is essential that the presence of such molecules in the environment be taken into consideration for future design and operation of catalytic microjet engines. We show this effect on catalytic micromotors prepared by both rolled-up and electrodeposition approaches, demonstrating that such poisoning is universal for Pt catalyzed micromotors. We believe that our findings will contribute significantly to this field to develop alternative systems or catalysts for self-propulsion when practical applications in the real environment are considered.

## Abstract 2

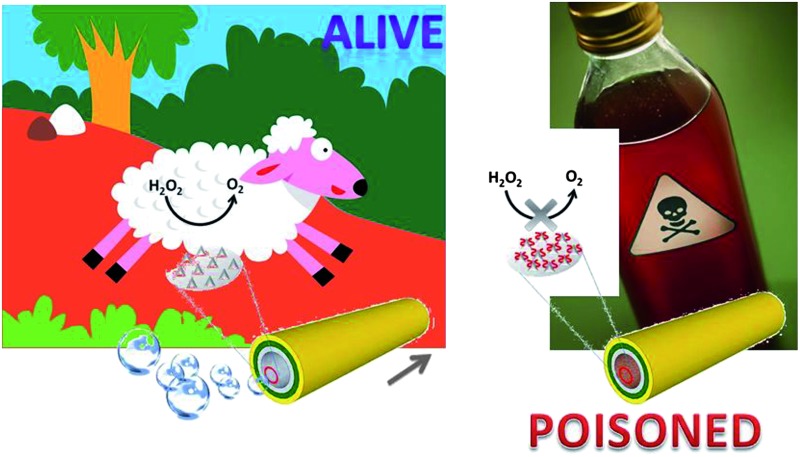
Sulfphur containing compounds, such as extracellular thiols (glutathione, cysteine, methionine) or organic solvents (DMSO) can cripple drastically the motion of Pt catalytic microjets even at very small concentrations.

## Introduction

**Scheme 1:**
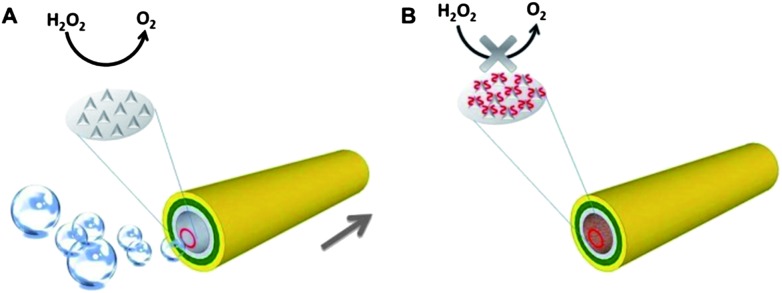
Poisoning of Pt-catalyst microjets with small molecules containing sulphur. (A) In the absence of the Pt-poisoning molecules, robust decomposition of H_2_O_2_ takes place in the tubular cavity, which is catalysed by the Pt surface of the cavity; (B) in the presence of Pt-poisoning molecules, the decomposition reaction is quenched or inhibited to a certain degree, fewer or no oxygen bubbles could be generated, leading to a lower or zero speed of the microjets.

In recent years, catalytic micro/nanomotors have attracted much interest amongst researchers.^1–7^ It is hoped that these micro/nanomotors can be operated autonomously in various natural environments, such as biological liquids and environmental waters, in order for them to perform critical tasks which can include microsurgeries,^8–10^ the discovery of natural resources^11^ or aid in environmental remediation.^12,13^ These catalytic micro/nanomachines can be powered by various mechanisms, such as self-electrophoresis, self-diffusiophoresis or bubble jet ejection.^3^ While the feasibility of the former two phoretic mechanisms has been demonstrated, the bubble-jet mechanism has recently been heavily investigated as it allows fast motion of the micro/nanodevices with a significant power output. The mechanism of the bubble-jet propulsion is rather simple: fuel in the form of hydrogen peroxide is catalytically decomposed to O_2_ in the inner Pt surface of the tubular microjet (refer to Scheme 1) where bubbles are asymmetrically ejected, propelling the micromotor in the opposite direction, allowing the jets to move not only at very high speeds,^14,15^ but also to perform highly powered tasks like the delivery of cargo,^16^ manipulation of multiple cells,^17^ isolation of biomolecules or cells,^18^ penetration of cells and tissues,^8–10^ as well as the removal of oil contaminants from the environment.^12^

However, when real-world applications are considered, one should keep in mind that the components of those environments may have a negative impact on the motion of the microjet engine. Pt as a catalyst is prone to poisoning mostly by sulphur-containing compounds. These compounds such as sulphur containing peptides and amino acids are present in blood and in cells. As for other sulphur containing compounds like sulphoxides, they may be present in environmental waters dedicated for remediation. To date, there is a lack of studies on the effect of environmental molecules on the Pt-catalyst micromotors. Most of the current research is focused on fabrication of more powerful nano/microengines^2,14,15^ or on demonstration of the proof of principle of the applications;^8–10,12,13^ however, the issue of negative influence of components of the environment on the movement of microjets is rarely taken into account.^19^

In this paper, we demonstrate that at a constant hydrogen peroxide concentration (9 wt%), the motion of the microjets can be significantly inhibited by chemicals present in the environment. Since the motion of the microjets depends on the rate at which oxygen bubbles are generated,^20^ the presence of certain molecules would have a significant effect on the motion if they can affect the reaction of hydrogen peroxide. Some organic and/or biological molecules are capable of quenching or inhibiting the H_2_O_2_ decomposition reactions catalysed by Pt. In this study, dimethyl sulfoxide (DMSO) was used to chemically quench the ˙OH radical generated in the decomposition of H_2_O_2_. In addition, several sulphur containing peptides and amino acids were also chosen to study the poisoning effect they have on the microjets since sulphur containing species (H_2_S, RSH, RSSR…) are known poisons for all catalytic processes using Pt-catalysts.^21^ With the presence of the –SH or –SCH_3_ moiety, the motion of the microjets was reduced in terms of speed and the number of microjets running (Scheme 1). It is also shown that a higher concentration would be required for the –SCH_3_ containing methionine molecules to affect the motion of the microjets, indicating that the Pt catalyst is much more sensitive to –SH moieties. Our results reveal that organic/biological molecules present in the natural/biological environment can dramatically inhibit the motion of microjet engines based on the Pt catalyst. This must be carefully considered when employing the microjet for real-world tasks.

## Results and discussions

In this paper, we demonstrate that certain biomolecules and simple organic molecules containing sulphur moieties can significantly inhibit the motions of the microjets. The inhibition of such motions can take place in two ways: (i) quenching of ˙OH radicals generated by the Pt catalysed disproportionation of H_2_O_2_, and (ii) the poisoning of the Pt catalyst surface. We will discuss these two cases in the following subsections. We first demonstrate this inhibition of movement of catalytic microjets prepared by rolled-up technology and consequently of microjets prepared by electrochemical deposition.

### Quenching of O_2_ gas generation with DMSO

With the presence of a catalyst (in this case, Pt metal), hydrogen peroxide decomposes in a disproportionation reaction to generate O_2_ gas. The reaction proceeds *via* a radical pathway.^22^ During the reaction, the hydroxyl radical ˙OH is the key to the formation of O_2_ gas bubbles.^23^ Based on the reaction mechanism, when the chemical quencher (DMSO in this case) is present, the rate of O_2_ gas generation is given by:^23^ 
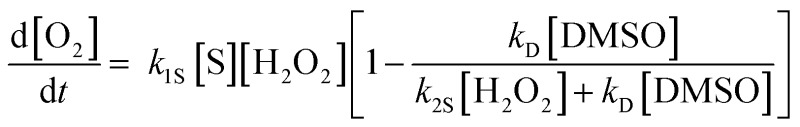
where [S] stands for the active metal surface, *k*_D_, *k*_1S_ and *k*_2S_ represent the constants in the reaction processes and [DMSO] and [H_2_O_2_] are the concentrations of DMSO and hydrogen peroxide in the solution, respectively. From the above equation, the generation of oxygen gas depends on three factors: the concentration of hydrogen peroxide, the amount of DMSO (or other molecules that are able to quench the radical reaction) as well as the active surface area of the catalyst metal, Pt.

Since the generation of O_2_ gas proceeds through the radical mechanism, the motion of the jets can be very sensitive to molecules that are capable of quenching the radicals. To run the jets in a chemically complex medium – which is the case if we use them in a natural sample for certain applications, it may be difficult to avoid the presence of such ˙OH radical-quenching molecules. In this study, DMSO was used as a model “˙OH radical quenching molecule” as it is a well-known quencher for the ˙OH radicals.^24^

**Fig. 1:**
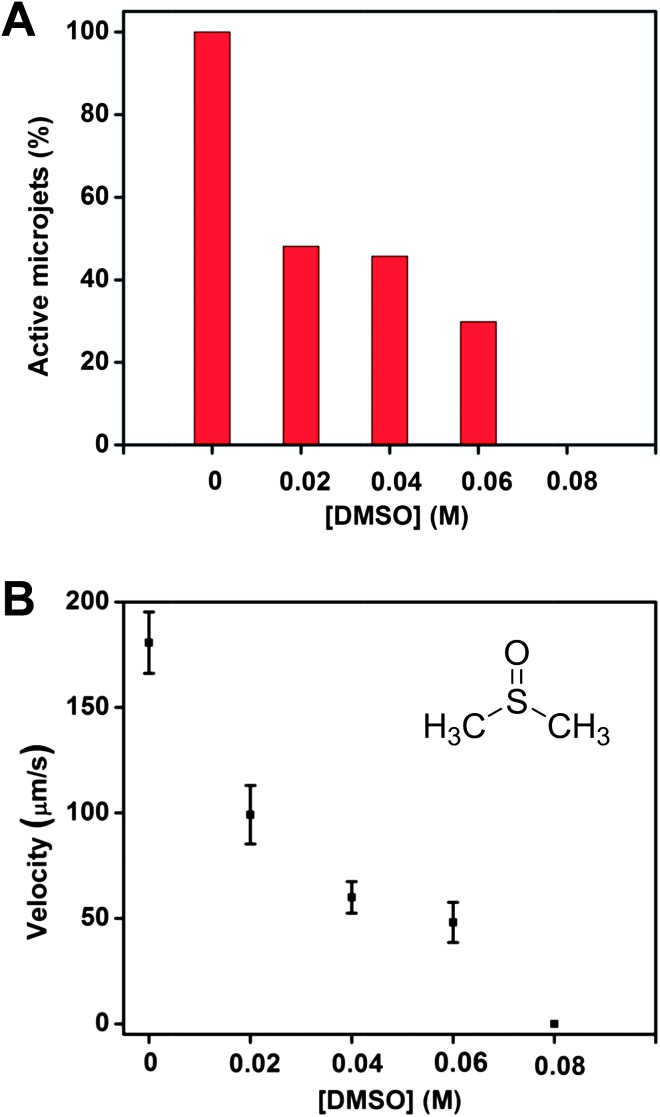
The presence of the DMSO in the running solution can significantly reduce the ability of catalytic microjets to move. (A) Influence of the DMSO concentration on the population of the microjet engines found to be exhibiting motion. Note that more than half of the jets stopped running at a low concentration of DMSO at 20 mM (running percentage in solutions without DMSO was defined as 100%), and no motion could be observed at all when the concentration of DMSO increased to 80 mM; (B) influence of the concentration of DMSO on the velocities of the microjets which exhibited non-zero velocity motion. Note that at 80 mM of DMSO, there was no motion observed for any microjet. Tracking data were obtained for a timescale of 10 seconds from 5 independent running experiments in order to get the average speed.

In an aqueous solution of 9 wt% of H_2_O_2_, we studied the influence of DMSO concentration on the motion of the microjets (if not mentioned otherwise, we discuss in the following text microjets prepared by rolled-up nanotech).^19^ Fig. 1 summarizes the findings. In the presence of 20 mM of DMSO, more than half (56.25%; *n* = 32, where *n* is the total number of jets counted) of the microjets showed absolutely no motion; they were completely deactivated by the quenching effect, see Fig. 1A. DMSO molecules are able to quench the radicals that were produced *in situ*, even at a high concentration of hydrogen peroxide (9 wt%; this corresponds to a ratio of ∼145 H_2_O_2_ molecules *vs.* 1 DMSO molecule). As the DMSO molecules are homogenously distributed in the solution; they are taken in by the microjet inlet together with the H_2_O_2_ fuel, quenching the radicals as soon as they are formed. When the concentration of the DMSO was increased to 80 mM, all the microjets were deactivated – in other words, no microjets showed any motion.

Other than the complete deactivation of a large percentage of the jets (or all of them at concentrations of ≥80 mM), the presence of DMSO also resulted in a significant decrease of the velocities of the microjets, among those which were still able to acquire motion, as shown in Fig. 1B. A significant reduction of the velocities was observed even with the presence of only 20 mM of DMSO. The average speed decreased from ∼180 to ∼99 μm s^–1^. This is due to the quenching of the ˙OH radicals which led to the decreased production of oxygen bubbles, and this in turn resulted in a weaker thrust in the motion of the jets.

### Poisoning of the Pt catalyst with extracellular thiols

It is well known that sulphur containing species (H_2_S, RSH, RSSR) act as poisons for catalytic processes which employ Pt and this may cause significant deactivation of the catalyst even at a very low concentration, due to the formation of strong metal–sulphur bonds.^21^ Sulfur is able to chemisorb onto the active sites of the catalyst, inhibiting the catalytically reactive sites. What is more, the possible non-selective side reactions can be triggered by the stable bonds between the metal and the adsorbate, and this can even modify the surface properties of the catalyst to a further extent.^26^

**Fig. 2:**
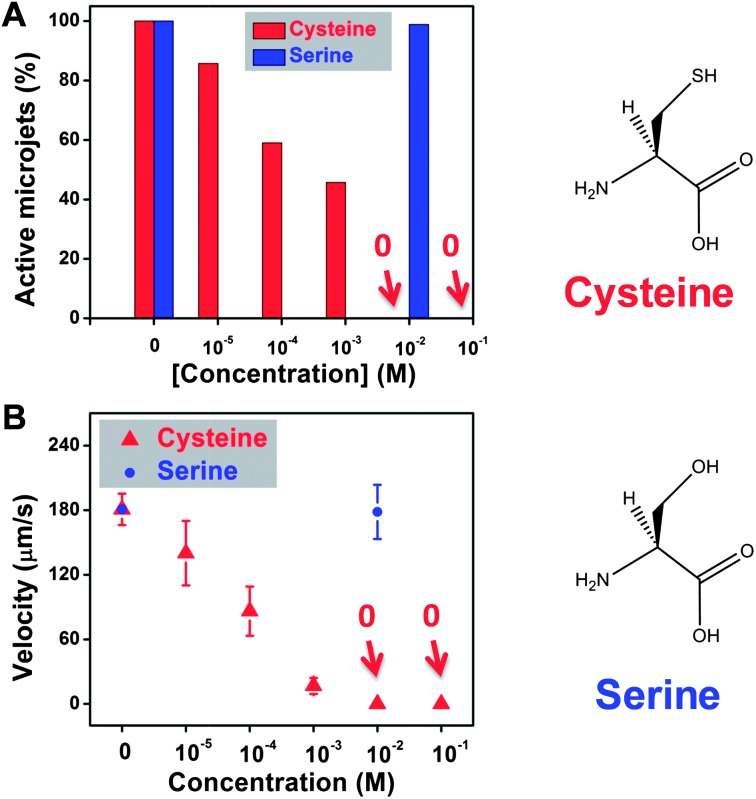
Presence of cysteine molecules in the running solution can significantly reduce the motion of the rolled-up microjets. (A) More than half of the jets stopped running at 0.001 M of cysteine concentration, and no more motion could be observed when the concentration increased to 0.01 M. Nearly 100% of the jets were found running in serine-containing solution; (B) significant reduction in speed was observed for the jets, from ∼180 μm s^–1^ without cysteine to ∼16.7 μm s^–1^ at 0.001 M concentration of cysteine. The velocity remained at ∼180 μm s^–1^ for jets running in serine-containing solutions at 0.001 M concentration. Tracking data were obtained for a timescale of 10 seconds from 5 independent running experiments in order to obtain the average speed.

Several extracellular thiols, such as cysteine, methionine and glutathione, are of important physiological significance. They exist in extracellular as well as in intracellular liquids, acting as signal transducers and antioxidants. The levels of these compounds present in biological fluids, such as plasma and urine are important biomarkers in various clinical situations and they are often present in concentrations of 1–10 mM.^27–30^ These thiols contain either an R–SH or R–S–S–R moiety. Hence it is of paramount importance to determine whether the presence of these extracellular thiols can poison the Pt interior of a microjet engine and hinder the catalytic conversion of H_2_O_2_ to oxygen bubbles, which would disable the motion of the microjets. We first investigated one of the simplest of these thiols, cysteine. We also investigated the reason behind the poisoning to determine if it is indeed the –SH group that is responsible, by using serine as a control additive to the solution. Serine has the same structure as cysteine with the exception of the side chain moiety, which is –SH for cysteine and –OH for serine. The cysteine molecules are readily oxidized by hydrogen peroxide to form a disulfide bond in the presence of hydrogen peroxide in the running solution.^31^ The presence of a disulfide bond and a possible residual –SH group in the running solution can function as poisoning agents for the catalytically active Pt metal surface,^21^ which is evident from Fig. 2. Two sets of experiments under the same conditions were carried out for cysteine-containing and serine-containing solutions. As shown in Fig. 2A, even in the presence of 10 μM (10^–5^ M) of cysteine, about 15% of the microjets were disabled, accompanied with a significant reduction of the velocity of remaining microjets. This is due to the coverage of the inner Pt surface of these microjets with an active R–SH group, which suppressed the disproportionation of H_2_O_2_. At 1 mM concentration of cysteine-containing solutions, only less than half of the microjets were found moving; and when the concentration was increased to 10 mM, none of the microjets were found running. In contrast, almost 100% of the microjets were found to be running for the serine-containing solution at 10 mM concentration. Furthermore, not only do the cysteine molecules “disable” the jets but they also reduce the power for the moving ones, in a similar manner to that of the DMSO molecules. The average speed in the first 10 seconds decreased from ∼180 μm s^–1^ to ∼86.1 μm s^–1^ at 0.1 mM of cysteine concentrations, and continued decreasing to 16.7 μm s^–1^ for 1 mM and eventually to 0 μm s^–1^ at 10 mM concentration of cysteine. In addition, the velocities of the microjets remained the same whether it is in the absence or presence of 10 mM of serine (∼180 μm s^–1^). As seen for the cysteine-containing solutions, partial deactivation of the active Pt catalytic sites led to a lower production of oxygen bubbles, resulting in a weaker thrust pushing the jets to move; hence a reduction in the velocities was observed. In order to prove that such decrease of microjet performance is indeed due to the –SH group present in cysteine, we used an analogue of cysteine, as a control experiment. Serine differs from cysteine only by the substitution of the –SH group by the –OH group. In comparison, such reduction of velocities was not observed for the serine-containing solutions. Since the difference between the structures of a cysteine and a serine molecule lies only in the –SH and –OH side-chain moiety, it is thus clear that it is indeed the –SH groups that react in the solution, and are responsible for the inhibition of the motion of the microjets.

**Fig. 3:**
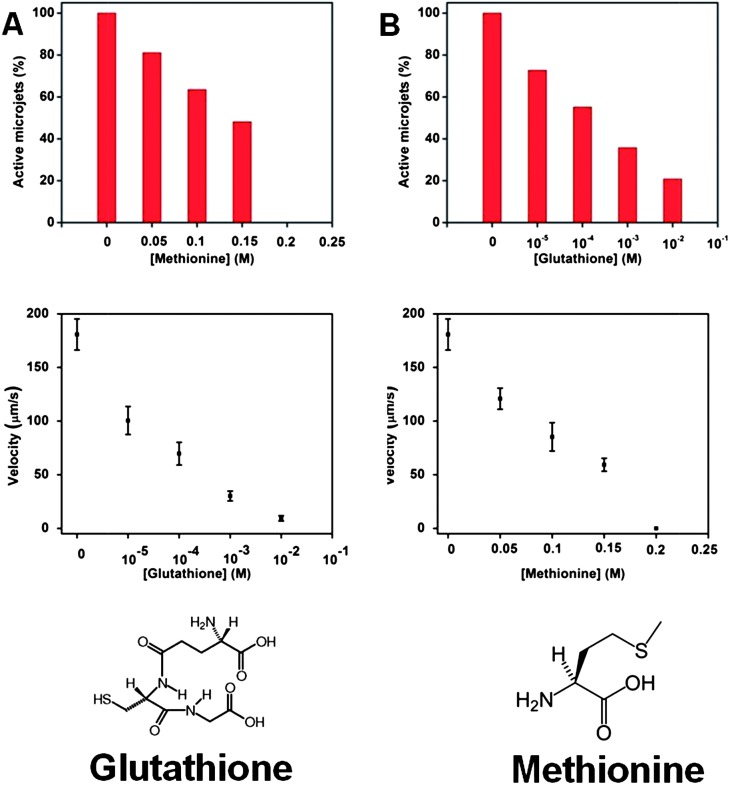
Comparison of the poisoning effect for glutathione and methionine molecules in the running solution (A) less than 40% of rolled-up tubes were running with only 0.001 M of glutathione present in the solution, and a significant reduction of speed was also observed for the jets, from ∼180 μm s^–1^ without glutathione to ∼30 μm s^–1^ at 0.001 M concentration of glutathione; (B) more than half of the jets stopped running at 0.15 M methionine concentration and no more motion could be observed when the concentration increased to 0.2 M, and a significant reduction of speed was also observed for the jets, from ∼180 μm s^–1^ without methionine to ∼60 μm s^–1^ at 0.15 M concentration of methionine; tracking data were obtained for a timescale of 10 seconds from 5 independent running experiments in order to obtain the average speed.

Once we have proved that the –SH group is responsible for the poisoning the microjet engine, we proceeded to investigate more complex thiols. Glutathione is a very important tri-peptide which contains the –SH moiety (for the structure of glutathione, see Fig. 3), and it is responsible for its anti-oxidative function, present in ∼5 mM concentrations in the mammalian cells.^27,28^ The Pt catalytic microjets were found to be very susceptible to poisoning by glutathione at a concentration much lower than the physiological concentrations of this tri-peptide. At 0.1 mM (10^–4^ M) concentration, almost half of the microjets were disabled (exhibited no motion) and the velocity of the remaining moving jets was dramatically reduced from ∼180 to ∼70 μm s^–1^ (Fig. 3A). At the physiological concentrations of glutathione, which are in the 1–10 mM range, about 65–80% of jets were disabled and the remaining exhibited a crippled motion at 10–30 μm s^–1^ (compared to ∼180 μm s^–1^ in the absence of glutathione).

Other than the R–SH and RS–SR′ moiety, the R–SR′ moiety is also able to poison the Pt catalyst. As shown in Fig. 3, nearly half of the jets were “disabled” at 150 mM of methionine. It is evident that the R–S–CH_3_ moiety of methionine is less poisoning than that of the R–SH moiety in cysteine (note that the R portion of a molecule of cysteine and that of methionine possess very close structures) and in glutathione, even though it is still strongly poisoning the Pt catalytic microjets at ∼100 mM concentration levels.

This is due to the fact that methionine (in contrast to cysteine) does not contain the –SH moiety but only the –SCH_3_ moiety, which exhibits a weaker bond to Pt (*via* chelation), in a similar manner as in methionine platinum dichloride.^32^

**Fig. 4:**
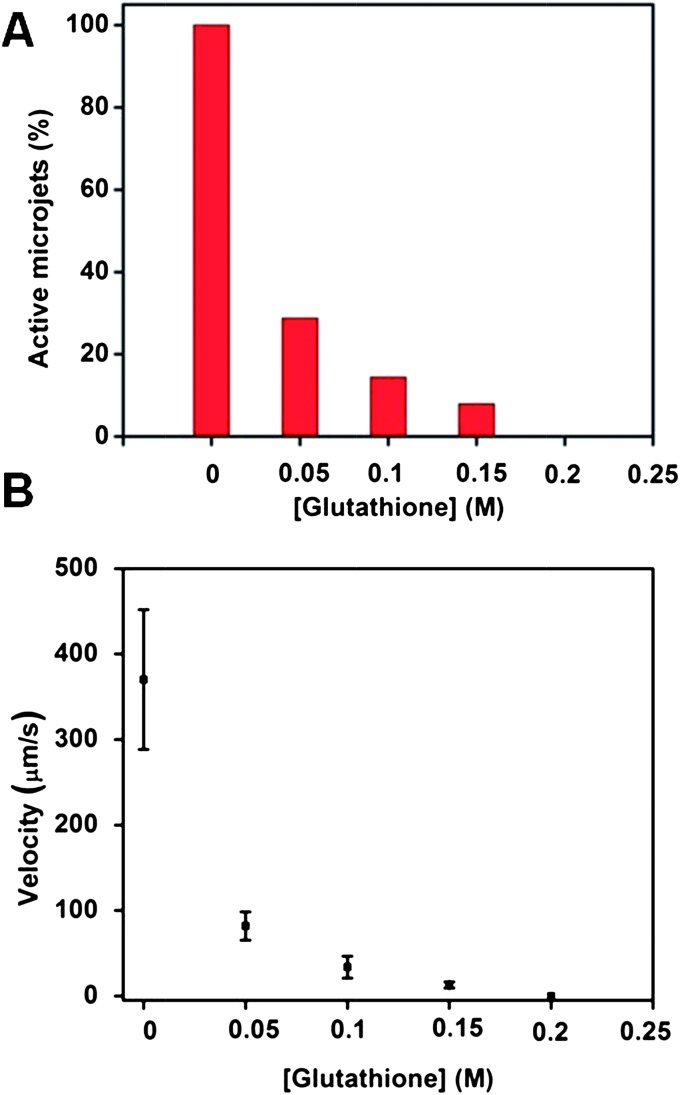
The presence of glutathione in the running solution can reduce the ability of Cu/Pt catalytic microjets prepared by electrodeposition to move. (A) Influence of the glutathione concentration on the population of the microjet engines found to exhibit motion. No motion could be observed at all when the concentration of glutathione increased to 200 mM; (B) influence of the concentration of glutathione on the velocities of the microjets which exhibited non-zero velocity motion. Tracking data were obtained for a timescale of 10 seconds from 5 independent running experiments in order to obtain the average speed.

In order to show that the above-mentioned effects do not influence only the catalytic microjets prepared by rolled-up nanotech,^25^ we performed similar experiments on microjets of smaller dimensions prepared by electrochemical deposition. These microjets consisted of microtubes with a Pt interior, with a length of ∼10 μm and a diameter of ∼2 μm (compared to the size of rolled-up microtubes with a length of ∼50 μm and a diameter of ∼5 μm). Not surprisingly, these microjets behaved similarly to the ones prepared by rolled-up technology which we described in the previous sections. More specifically, microjets made from template-assisted electrodeposition were also found to be very susceptible to being poisoned by glutathione. At 50 mM (0.05 M) concentration of glutathione, more than 70% of the microjets were disabled (exhibited no motion) and the velocity of the remaining moving jets was dramatically reduced from ∼380 to ∼80 μm s^–1^ (Fig. 4). The mechanism of poisoning is the same as in the case of rolled-up microtubes.

## Conclusion

In this study, we have established that typical organic and/or biological molecules can significantly reduce the motion of Pt catalysed microjet engines. DMSO molecules were able to hamper the running of the jet engines by quenching the hydroxyl radicals and reducing the amount of oxygen gas generated in the decomposition of hydrogen peroxide molecules. Extracellular thiols, such as cysteine, methionine and glutathione with sulphur-containing moieties can also poison the catalytically active site on the Pt surface. Most of the biological liquids contain these extracellular thiols, often in millimolar concentrations and at these concentrations, the microjets movement has been proven to decrease dramatically. Therefore, the presence of such molecules in the chosen running media must be considered for the future design and operation of catalytic microjet engines in the real-world applications.

## Experimental section

### Materials

The cyclopore polycarbonate membranes with pores of 2 μm diameter were purchased from Whatman, USA (Cat no. 7060-2511). The pores are conical shaped. Colloidal graphite (isopropanol base) was purchased from Ted Pella, Inc. (Lot no. 12009-2, USA). Hydrogen peroxide (27%, Lot no.: 10151507) was purchased from Alfa Aesar, Singapore. Dimethyl sulfoxide (DMSO), methylene chloride and ethanol were purchased from Tedia, USA. Pt electrodes with 1 mm diameter and Ag/AgCl/1 M KCl were purchased from CH Instruments, USA. CuSO_4_·5H_2_O (98+%) was purchased from Sigma-Aldrich. The platinum plating solution (Lot no. 20251001) was purchased from Technic Inc., USA. Sodium dodecyl sulfate (SDS, Lot no. 079K0335), l-cysteine, l-serine, l-methionine and l-glutathione reduced were purchased from Sigma-Aldrich. Chemicals were used as received and the solutions were prepared using ultrapure water (18.2 MΩ cm) from a Millipore Milli-Q purification system.

### Apparatus

Electrochemical deposition was carried out with a μAutolab type III electrochemical analyzer (Eco Chemie, The Netherlands) connected to a computer and controlled by General Purpose Electrochemical Systems version 4.9 software (Eco Chemie). The deposition procedure was conducted at room temperature (25 °C) using a three-electrode arrangement. A platinum electrode was utilized as a counter electrode, and Ag/AgCl was used as the reference electrode. The ultrasonication process was carried out with a Fisherbrand FB 11203 ultrasonicator, and centrifugation was carried out with a Beckman Coulter Allegra 64R centrifuge. Scanning electron microscopy (SEM/EDX) analysis was obtained with a JEOL JSM 7600F instrument. Optical microscope videos and images were obtained with a Nikon Eclipse TE 2000-E microscope. Video sequences (100 fps) were processed with Nikon NIS-Elements software.

### Methods

#### Preparation of rolled-up microtubes

Ti/Fe/Cr/Pt microtubes were fabricated by electron-beam (e-beam) evaporation (Ti/Fe/Cr) and magnetron sputtering (Pt) of thin metallic films on patterned photoresist squares with a single element size of 50 μm × 50 μm. Photoresist AR-P 3510 was spin coated on silicon wafers (1.5 inch) at 3500 rpm for 35 s, followed by a soft bake using a hotplate at 90 °C for 1 min and exposure to UV light with a Karl Suss MA-56 mask aligner (410–605 nm). Photoresist patterns were then developed in an AR300-35/H_2_O solution (1 : 1). Rolled-up catalytic microtubes were obtained by a tilted deposition at an angle of about 60° (measured from the horizontal axis) on the photoresist. Metallic particle films (Ti/Fe) with thicknesses of 3 nm (Ti), 5 nm (Fe) and 5 nm (Cr) were deposited layer by layer on the tilted samples. Thereafter, by using magnetron sputtering, 1 nm of Pt was deposited onto the Ti/Fe/Cr samples. The samples were immersed in acetone where rolling-up of the thin metallic films into microtubes was achieved by selectively etching the photoresist layer. Supercritical point drying was used in order to avoid collapsing of the tubes because of high fluid surface tension.^16^

#### Preparation of Cu/Pt concentric bimetallic microtubes

The Cu/Pt concentric bimetallic microtubes were synthesized with a modified electrochemical deposition procedure on a cyclopore polycarbonate template. Colloidal graphite ink was applied on one side of the polycarbonate template with commercial cotton swabs. A piece of flattened aluminium foil was attached to the ink immediately, which serves as the working electrode for the plating experiments. The template was assembled into a customized electrochemical deposition cell. Platinum counter electrode and Ag/AgCl reference electrode were utilized. Electrochemical deposition was carried out with a μAutolab type III electrochemical analyser connected to a computer and controlled by General Purpose Electrochemical Systems version 4.9 software. The template was rinsed with 5 mL of ultrapure water (18.2 MΩ cm) for 4 times, and the Cu outer layer was deposited galvanostatically at –4 mA for 450 s. The deposition solution contained 1 M CuSO_4_. Consequently, after removing the solution, the template was rinsed 5 times with 8 mL of water. The platinum segment was electrodeposited subsequently at –4 mA for 450 s each, using the commercial plating solutions. When the deposition of microtubes was finished, the electrochemical cell was disassembled and the template was washed for 5 times with 8 mL of water each. After that, the template was ultrasonicated for 3 times in 2 mL of ultra-pure water for 3 min each time. The graphite layer was removed during the sonication procedure. The template was placed in an Eppendorf tube with 2 mL of methylene chloride and ultrasonicated until the whole template dissolved. The electrochemically deposited microtubes were collected by centrifugation at 6000 rpm for 3 min and washed repeatedly for 3 times with methylene chloride. The solution was then washed with ethanol and water for two times each and centrifuged for 3 min after each washing step. The tubes were stored in water at room temperature.^33^

### Poisoning effect study

The experiments for the study of the poisoning effect were carried out in an aqueous solution containing 9 wt% of hydrogen peroxide for the rolled-up microtubes and 2 wt% of hydrogen peroxide for the electroplated microtubes at constant surfactant concentrations (1 wt% of SDS). A mixture of microjets (5 μL), SDS (1 wt%), H_2_O_2_ (9 wt% for rolled-up tubes and 2 wt% for electroplated tubes) and various concentrations of “poisonous” molecules were applied on a glass slide freshly cleaned with nitrogen gas. Optical microscope videos and images were obtained with a Nikon Eclipse TE 2000-E microscope. Video sequences (100 fps) were processed with Nikon NIS-Elements™ software. For each of the data points of Fig. 1, 2, 3 or 4, at least 32 measurements were taken into account.
